# Performance of Indocyanine Green Compared to 99mTc-Nanocolloids for Sentinel Lymph Node Detection in Early Vulvar Cancer

**DOI:** 10.3390/curroncol29110638

**Published:** 2022-10-26

**Authors:** Camélia Benmoulay-Rigollot, Georgia Karpathiou, Nathalie Prevot-Bitot, Mellie Heinemann, Beatrice Trombert-Paviot, Tiphaine Barjat, Céline Chauleur

**Affiliations:** 1 Department of Gynecology and Obstetrics, University Hospital, 42055 Saint Etienne, France; 2 Department of Pathology, University Hospital, 42023 Saint Etienne, France; 3 Department of Nuclear Medicine, University Hospital, 42023 Saint Etienne, France; 4 Department of Gynecology, LEON BERARD Center, 69008 Lyon, France; 5 INSERM U1059, 42023 Saint Etienne, France

**Keywords:** sentinel lymph node, vulvar cancer, radioactive, colorimetric, intraoperative detection

## Abstract

Study objective: The aim of this study was to evaluate the performance of indocyanine green (ICG) compared to that of the gold standard 99mtechnetium (99mTc-nanocolloids) in detecting sentinel lymph nodes (SLN) in early vulvar cancer. Material and Methods: A single-center retrospective cohort study comparing SLN detection by 99mTc-nanocolloids and ICG was performed in patients presenting early vulvar cancer (T1/2), with clinically negative nodes. All SLN showing a radioactive and/or fluorescent signal were resected. The primary endpoints were the sensitivity, positive predictive value (PPV) and false negative (FN) rate of ICG in detecting SLN compared to 99mTc-nanocolloids. Results: Thirty patients were included and 99 SLN were identified in 43 groins. Compared to 99mTc-nanocolloids, ICG had a sensitivity of 80.8% (95% CI [72.6; 88.6%]), a PPV of 96.2% (95% CI [91.8; 100%]) and a FN rate of 19.1% in detecting SLN. Seventeen (17.1%) infiltrated (positive) SLN were identified out of the 99 SLN detected. Compared to 99mTc-nanocolloids, ICG showed a sensitivity of 82.3% (95% CI [73.1; 91.5%]), a PPV of 100% and a FN rate of 17.6% (3/17) in detecting infiltrated SLN. Conclusion: Despite its many advantages, ICG cannot be used as the sole tracer for the detection of SLN in early vulvar cancer and should be employed in conjunction with 99mTc-nanocolloids.

## 1. Introduction

Vulvar cancer is a rare cancer with an incidence of 0.5 to 1.5/100,000 women in France [1]. It represents 5% of all gynecologic cancers and is the fourth most prevalent gynecologic cancer worldwide [2,3]. Inguinal lymph node (LN) status is the major prognostic factor for vulvar cancer [4,5]. From a depth of invasion greater than 1 mm (FIGO IB), the risk of LN infiltration rises to 35% [6], and LN exploration is essential. Up to 1990, inguinofemoral lymphadenectomy (IFL) was performed systematically to assess LN status in vulvar cancer. This radical dissection caused significant morbidity and was unnecessary in 70% of patients with stage I or II vulvar cancer [7,8]. Surgical practices have therefore changed in recent years, sentinel lymph node (SLN) identification having emerged as an alternative to IFL. If this first LN group is free of metastasis, the risk of distant dissemination is minimal [9]. Several studies have shown that use of the SLN procedure does not lead to any increased risk in vulvar cancer and reduces morbidity [9,10,11,12,13].

These advantages have led to recommendation of the SLN procedure in vulvar cancers less than 4 cm in diameter. However, in the event of SLN identification failure, ipsilateral IFL is indicated [1,8]. It is therefore essential to detect the SLN as precisely as possible since their analysis will determine subsequent therapeutic management [14]. The combination of radioactive and colorimetric markers for the detection of SLN is currently recommended [1]. SLN detection rate and sensitivity are increased by the use of both a radiotracer and patent blue compared to use of the isotopic or colorimetric method alone. This dual procedure avoids SLN detection failures and consequently recourse to IFL [15,16]. The radioactive tracer used in vulvar cancer is a radiolabeled colloid containing 99mtechnetium (99mTc) permitting pre-operative mapping of the lymphatic drainage of the tumor and estimation of the number of SLN [17]. Use of a gamma probe allows intraoperative detection of SLN. When employed alone, 99mTc-nanocolloids achieve a SLN detection rate of 95% [18]. However, this tracer provides solely acoustic feedback and, in addition to its high cost, its use exposes the patient to ionizing radiation.

Historically, patent blue was the standard colorimetric marker associated with 99mTc-nanocolloids. This dye has the advantage of being easy to use, marking the lymphatic ducts in blue and facilitating dissection of the groin. The SLN detection rate is 68.7% with the use of patent blue alone, increasing to 97.7% with the use of patent blue combined with 99mTc-nanocolloids [18]. The use of patent blue nevertheless has drawbacks. The main risk is the occurrence of severe allergic reactions in 0.2% of cases and anaphylactic shock in 0.1% of cases [19]. Furthermore, patent blue-labeled SLN are not visible intraoperatively through the skin or through fatty tissue. After skin incision, the surgeon’s vision may be reduced in the event of section of a lymphatic duct resulting in flooding of the surgical site with patent blue. These factors have led several teams to abandon colorimetric SLN detection in favor of isotopic detection alone.

In recent years, indocyanine green (ICG) has emerged as a new oncologic marker, improving SLN detection in breast, cervical and endometrial cancers [9,15,17,20,21]. ICG is considered to be an inert agent [22,23]. It is a fluorescent compound that emits light in the near-infrared (NIR) band (600–900 nm). Its transcutaneous migration can be traced to a depth of 15 mm. The advantages of ICG are better tissue penetration, transcutaneous visualization of lymphatic vessels and real-time visual guidance [24,25]. Few teams have investigated ICG for the detection of SLN in early vulvar cancer [26,27,28,29,30,31,32,33,34], but the studies performed have demonstrated that it is reliable and effective for this purpose.

Given its many advantages, this promising marker seems to be the optimal colorimetric agent for use in combination with the radiotracer 99mTc-nanocolloids. We therefore evaluated the performance of ICG compared to 99mTc-nanocolloids (the gold standard) in terms of sensitivity, positive predictive value (PPV) and false negative (FN) rate in the detection of SLN in early vulvar cancer.

## 2. Materials and Methods

This was a single-center, retrospective, cohort study carried out in the University Hospital of Saint-Etienne. The study was approved by the local ethics committee. The study included patients having undergone surgery for vulvar cancer diagnosed either on a preoperative biopsy specimen or on pathological examination of the vulvectomy specimen.

The search for SLN could have been carried out at the same time as vulvectomy or as a second step when the final pathology results showed an infiltrating tumoral contingent. Patients aged over 18 years presenting vulvar cancer of any histological type except sarcomas at an early clinical and radiological stage (T1-T2, N0, M0) were eligible for inclusion. Patients for whom dual SLN detection by TC-nano-colloids and ICG had not been envisaged, as well as pregnant or breastfeeding women and women allergic to ICG, were excluded. Planned IFL was an exclusion criterion. However, an exception was made for patients undergoing unilateral IFL due to suspect palpable adenopathy detected during surgery in whom contralateral SLN biopsy had been envisaged initially. As part of our tumor registry, all patients had given signed consent for the possible use of their clinical and pathological data in a study.

The SLNs were identified in the nuclear medicine department either on the day before surgery or at the same day. The procedure consisted of an intradermal peritumoral injection of either colloidal rhenium sulfide labeled with 99mtechnetium or nano-colloids of human serum albumin (HSA) labeled with 99mTc-nano-colloids. The static planar images obtained by lymphoscintigraphy under a gamma camera allowed mapping of the lymphatic drainage system, determination of its unilateral or bilateral nature and estimation of the number of SLN.

ICG was injected by the surgeon under general anesthesia in the operating room, after skin disinfection and before starting the vulvectomy procedure. The ICG ampoule contained 10 mL with 25 mg. After dilution in 10 mL of glucose serum, the ICG was injected near the vulvar lesion, at 2 points, subcutaneously or superficially, 1 mL then 1 mL deeper, at a rate of 2 mL, or 2.5 mg per point. The first transcutaneous lymphatic drainage relay was visualized 10 min after the injection of ICG. SLN was unilateral if the tumor was lateral and bilateral if the tumor was located less than 1 cm from the midline. The approximate position of the SLN was identified with the aid of a standard gamma probe and the skin mark. No distinction between superficial or deep LN regarding the cribriform fascia was made. The surgeon began by searching for NIR-fluorescence using a Photodynamic Eye NOVADAQ camera. The fluorescent SLN was identified by following a fluorescent lymphatic channel, isotopic activity then being checked using a gamma probe. All fluorescent nodes were resected even if no radioactive signal was detected. If palpable 99mTc− and ICG− lymph nodes were identified that were not sufficiently suspect (<2 cm in size) to justify IFL, these were excised, in addition to the radioactive and/or fluorescent LN, to enable a definitive pathological analysis. If no SLN was found in a groin, this was regarded as a failure of SLN identification and IFL was performed according to French public hospital and European (ESGO) recommendations. All LN removed were sent for pathological analysis. No frozen section was performed. The pathological analysis of the sampled SLNs was performed according to ESGO recommendations. Positive SLN (SLN+) comprised either micro-metastases or macro-metastases. Negative SLN (SLN−) were considered healthy. No isolated tumor cells in SLNs were recorded.

Our primary objective was to assess the performance of ICG for the identification of vulvar SLN. This was evaluated by calculating the sensitivity, the PPV and the FN rate, defined by the number of 99mTc+/ICG− SLN. For our purposes, 99mTc was considered as the gold standard for SLN identification. All data were analyzed using SAS V9.4 statistical software.

## 3. Results

Between December 2014 and May 2020, 53 patients underwent surgery for vulvar cancer. Thirty patients were included in the study, 23 patients in the initial population selected being excluded owing to the absence of LN detection procedures for the following reasons: presence of an intraepithelial lesion or non-curative surgery with patient refusal of adjuvant treatments (*n* = 13), LN involvement detected at the time of clinical assessment and/or preoperative radiology (*n* = 5) and absence of dual detection of SLN by ICG and 99mTc-nanocolloids (*n* = 5). The clinical and tumor characteristics of the patients cohort are shown in Table 1. Two patients were classified as T2 owing to vaginal invasion revealed by the definitive pathological analysis. The final histological examination revealed a squamous cell carcinoma in 24 patients, a condylomatous carcinoma in two patients, a squamous cell carcinoma of the Bartholin gland in one patient and a melanoma in three patients. A total of 43 groins were explored in 30 patients.

The reasons for resorting to IFL were failure to detect unilateral SLN when there was an indication of bilateral SLN (1/30 patients) and discovery during surgery of palpable and suspicious lymphadenopathy in one of the two LN areas (2/30 patients); 5/30 patients included underwent SLN resection in a second operation, after initial vulvectomy and tumor excision.

Lymphoscintigraphy identified 53 SLN in 43 groins (Table 2). 99mTc-nanocolloids migrated unilaterally in one patient, necessitating IFL. This procedure led to the identification of four non-sentinel LN, all 99mTc− and ICG−, not infiltrated according to their pathological analysis and not included in the statistical analysis of this study. In two patients, although planar lymphoscintigraphy did not detect any SLN, several 99mTc+ SLN were identified during surgery.

**Figure 1:**
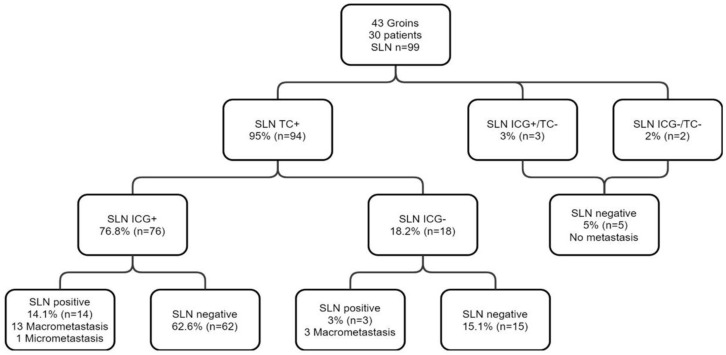
Flow chart presenting the results of SLN biopsy using both 99mTc and ICG in 30 patients with early vulvar cancer.

Pathological analysis identified a total of 99 SLN (Figure 1), of which 94 were 99mTc+. Three 99mTc−/ICG+ SLN were resected from two patients. These nodes were not located in isolation and were excised together with the 99mTc+/ICG+ SLN identified in the groins. Two palpable 99mTc−/ICG− LN were excised from two patients.

The sensitivity of ICG was 80.8% (95% CI [72.6; 88.6%]; Table 3). The PPV of ICG for SLN detection was 96.2% (95% CI [91.8; 100%]). Eighteen SLN were not detected by ICG, while being 99mTc+, corresponding to a FN rate of 18.2% (Table 3).

The 18 99mTc+/ICG− SLN concerned 12 patients (40%). A total absence of ICG migration was evident in six of the 30 patients included (20%) and a partial absence of ICG migration in the remaining six patients (20%). Seven of the 30 patients presented one or more infiltrated SLN, pathological analyses showing macro-metastases except in one patient with a micro-metastasis. A total of 17 SLN (17.1%) were infiltrated out of 99 detected. The 99mTc−/ICG+ (3/99) and 99mTc−/ICG− (2/99) nodes were not infiltrated. The sensitivity of ICG in the detection of SLN+ was 82.3% (95% CI [73.1; 91.5%]). The ICG PPV for SLN+ detection was 100%. There were three FN, reflecting failure to detect three infiltrated SLN with ICG in two patients. A complete absence of ICG migration was noted in one patient with two 99mTc+/ICG− SLN infiltrated. In the second patient, fluorescence was detected in the majority of the seven infiltrated SLN, comprising six 99mTc+/ICG+ and one 99mTc+/ICG−. In the sub-group of patients who had previous vulvar surgery, the sensitivity of ICG was 87.5%. The ICG PPV for sentinel lymph node detection was 93.3%.

## 4. Discussion

With regard to sensitivity, the performance of ICG appears to be insufficient compared to 99mTc in detecting SLN in patients with vulvar cancer. In our study, we found a sensitivity of 80.8% and a detection rate of 76.8% with ICG compared to the gold standard, 99mTc-nanocolloids. Several studies [26,27,28,29,30,31,32,33,34] have demonstrated the feasibility of intraoperative detection of the SLN by NIR-fluorescence using ICG in women with early vulvar cancer (Table 4).

The difference between our results and these studies, showing detection rates ranging from 89.7% to 100%, can be explained by the inclusion of all patients starting from the initial introduction of dual detection by 99mTc-nanocolloids and ICG, even before standardization of this bimodal detection technique. Furthermore, our protocol consisted in injecting 4 mL of ICG at two points whereas in the majority of studies, the ICG was injected at four peritumoral points at concentrations varying according to the study. Some studies showed a lower sensitivity of ICG compared to 99mTc-nanocolloids in the detection of SLN. Crane et al. [29] found a correlation between BMI and the rates of detection of SLN by NIR-fluorescence. In that study, transcutaneous fluorescence was visible in patients with a mean BMI of 25.6, whereas in patients with a BMI exceeding 28.8, SLN were not detectable by NIR-fluorescence prior to their identification by their radioactivity. Detection of fluorescent LN may be limited to a maximum depth of around 5 mm, although the precise cut-off may be higher or lower depending on the optical properties of the surrounding tissue [20]. In contrast, Prader et al. [32] concluded that there was no correlation between SLN detection rates and BMI. Furthermore, a review of published data showed a difference in the SLN detection rate with ICG depending on whether the analysis was performed in vivo or ex-vivo. Mathéron et al. [31] detected 96% of fluorescent SLN in vivo, whereas ex-vivo analysis revealed that all radioactive SLNs were also fluorescent.

Regarding the other parameters of our study, the PPV was 96.2%, two patients presenting three 99mTc−/ICG+ SLN, corresponding to 3% of the total number of SLN detected; these lymph nodes were not infiltrated. This low rate may correspond to false positives with respect to ICG or false negatives with respect to 99mTc-nanocolloids. Soergel et al. [26] similarly obtained a PPV of 91.9%. This finding could be explained by the lymphatic drainage of the ICG by other non-sentinel LN due to the prolonged interval between the injection of ICG and excision of the SLN. None of these LN was infiltrated. Broach et al. [33] studied 160 patients, exploring a total of 265 groins using different tracers. In the 99mTc plus ICG subgroup, 100% of SLN were detected. 99mTc-nanocolloids failed to detect the SLN in 14 patients, signifying that ICG had migrated alone in 14.6% of patients in the 99mTc plus ICG group. In the ICG subgroup, 96.3% groins were explored successfully, with a single failure of ICG to migrate into a groin.

The FN rate in our study was 19.1%. Levenback et al. [9] argued that this rate decreased with the experience of the surgeon, pathologist and nuclear physician. Prader et al. [32] found lower SLN detection rates both with ICG and with 99mTc-nanocolloids in the first year, but then observed an improvement with successful detection of SLN. The records showed failure to detect unilateral SLN using the combined tracer method in only a single patient in whom the procedure had been completed by IFL. This patient had undergone previous surgery of the vulva, which could explain the modification in lymphatic drainage. A meta-analysis reported by Hassanzade et al. [15], showed that SLN detection rates were lower in patients who had previously undergone vulvar surgery than in those without such a precedent, but this difference was not significant. Crosbie et al. [35] similarly reported a higher detection rate of SLN in patients with an existing tumor than in those with a history of vulvar surgery. In our study, the mean number of SLN detected per groin was the same irrespective of whether the tumor had been excised. ICG correctly assessed lymph node status in all but one (3.3%) of the 30 patients analyzed. This rate was consistent with the FN rate in the detection of SLN [13]. In view of these results, use of ICG should be coupled with that of 99mTc, as specified in the current French recommendations [1].

The aim of the treatment in vulvar cancer today is to reduce the radicality and morbidity of this surgery [36], and several procedures have changed to induce a minimally invasive surgical approach, especially by introducing SLN biopsy, nicely presented in recent works [37]. Our study is one of the largest cohort studies performed to date, including 30 patients with a total of 99 SLN identified and evaluating a promising method of SLN detection. Its limitations comprise bias due to its retrospective nature and the collection of data from computerized files, so we cannot exclude data omission.

## 5. Conclusions

Despite its many advantages, ICG cannot be used alone and should be coupled with 99mTc-nanocolloids for the detection of SLN in patients with early vulvar cancer. In centers lacking nuclear medicine facilities, it would be of interest to know whether ICG might represent a promising technique in the future. Prospective, randomized, multicenter studies are warranted to achieve this objective. Isotope detection is an expensive method compared to NIR-fluorescence imaging techniques and although these techniques require the purchase of an HD camera, this can be used in many other types of surgery.

## Figures and Tables

**Table 1:** Clinical and tumors’ characteristics.

Number of Patients (*n*)	30
Age (years) (Mean ± SD)	70.2 ± 11.2
Body Mass Index (Mean ± SD)	26.2 ± 7.2
Smokers, *n* (%)	4 (13.2%)
Diameter of tumor (largest axis in mm) (Mean ± SD)	29 ± 12.2
Depth of invasion (mm) (Mean ± SD)	6.2 ± 5.4
Tumor, *n* (%)	
Unifocal	26 (86.2%)
Multifocal	4 (13.2%)
Stage TNM, *n* (%)	
T1a	4 (13.2)
T1b	21 (70)
T2	2 (6.2)
Melanoma (1T1b and 2 T4b)	3 (10%)
Associated Lesion, *n* (%)	
HPV associated	6 (20%)
Lichen sclerosus	18 (60%)
Not found	6 (20%)
Location of primary tumor, *n* (%)	
Midline	18 (60%)
Lateral (right or left)	12 (40%)
Local treatment, *n* (%)	
Local excision + SLN procedure	25 (83.3%)
Wide excision + SLN procedure	3 (10%)
Only SLN procedure	5 (16.7%)
Inguinal surgery procedure, *n* (%)	
Unilateral SLN	14 (46.7%)
Bilateral SLN	13 (43.3%)
Unilateral SLN + IFL contro-lateral	3 (10%)

**Table 2:** Sentinel lymph nodes detected and excised.

Number of SLN detected by Lymphoscintigraphy, *n* (%)	53
Unilateral	22 (41.5%)
Bilateral	31 (58.5%)
Number of SLN identified by histology examination, *n* (%)	99 (100%)
Number of SLN excised, *n* (%)	
Unilateral exploration	39 (39.4%)
Bilateral exploration	60 (60.6%)
Intra-operatively detected SLN number per patient (Mean ± SD)	3.3 (±2.4)
Intra-operatively detected SLN number per groin (Mean ± SD)	2.3 (±1.5)
Detection modality, *n* (%)	
Radioactivity	94 (94.9%)
Fluorescence	79 (79.8%)
Radioactivity or and fluorescence	76 (76.8%)
Status of SLN, *n* (%)	
Negative	23 (76.7%)
1N+	4 (13.3%)
2N+	1 (3.3%)
>3N+	2 (6.6%)
Histology, *n* (%)	
Negative	23 (76.7%)
Micro-metastasis	1 (3.3%)
Macro-metastasis	6 (20%)

**Table 3:** Performance of the ICG in comparison with TC for the detection of SLN.

SLN	TC+	TC−	Total
ICG+	76 (76.8%)	3 (3%)	79 (79.8%)
ICG−	18 (18.2%)	2 (2%)	20 (20.2%)
Total	94 (95%)	5 (5%)	99 (100%)

**Table 4:** Characteristics of published studies and the different tracers used for SLN biopsy in patients with early vulvar cancer (ICG, patent blue and 99mTc).

First Author *	Year of Publication	*N* Patients	*N* Groins	*N* SLN	Tracers	ICG: Type, Concentration, Injected Points	Detection Rate with ICG
Crane	2010	10	16	29	ICG-PB, TC	ICG 0.5 mg/mL 4 Quadrants	ICG (26/29SLNB) 89.7%
Hutteman	2012	9	12	14	ICG-HSA, PB, TC	ICG−HSA 500, 750 or 1000 uM 1.6 mL 4 Quadrants	ICG-HAS (14/14SLNB) 100%
Schaafsma	2013	24	34	35	ICG-HSA, ICG, PB, TC	ICG+/−HSA 500 uM 1.6 mL 4 Quadrants	ICG+/−HSA (35/35SLNB) 100%
Mathéron	2013	15	27	46	ICG-TC, PB	ICG−TC 0.12 mg/mL 0.4 mL 3–4 Quadrants	ICG 96% In vivo 100% Ex vivo
Verbeek	2015	12	20	21	ICG-TC, PB	ICG−TC 161 uM 0.12 mg, 1 mL 3–4 Quadrants	ICG (21/21GS) 100% Ex vivo
Soergel	2017	27	52	91	ICG, TC, PB	ICG 1.25 mg/mL, 5 mL 4 Quadrants	ICG (91/91GS) 100% FP ICG = 8
Prader	2020	33	64	125	ICG, TC	ICG 1.25 mg/mL 2 mL peri-tumoral	ICG (117/125SLNB) 93.6%
Broach	2020	160	265	-	ICG+/-TC+/-PB	ICG -	ICG (96/96 groins)100% Failure TC 14.6%
Deken	2020	48	72	102	ICG-TC, TC+PB	ICG 161 uM 0.12 mg 4 Quadrants	ICG (49/53SLNB) 92.5%
Our Study	2020	30	43	99	ICG, TC	ICG 5 mg, 2 mL 1–2 Quadrants	ICG (76/99) 76.8% FP ICG = 3

SLNB = sentinel lymph node biopsy, ICG = indocyanine green, ^99m^Tc = ^99m^technetium, ICG-^99m^Tc hybrid = two tracers combined in a single injection, PB = patent blue, HSA = human serum albumin, FP = false positive. * = see references.

## Data Availability

Data are available upon reasonable request.

## References

[1] Uzan C., Canlorbe GMaingon P., Conforti R. Référentiel Cancer de la Vulve. Assistance Publique—Hôpitaux de Paris. https://www.google.com.hk/url?sa=t&rct=j&q=&esrc=s&source=web&cd=&ved=2ahUKEwjnjJH-ifH6AhUbQd4KHbeMAXUQFnoECBEQAQ&url=https%3A%2F%2Fwww.aphp.fr%2Fcontenu%2Freferentiel-cancer-de-la-vulve&usg=AOvVaw3woGWfy6Vv7xWhkNA73Gfy.

[2] Bodelon C., Madeleine M.M., Voigt L.F., Weiss N.S. (2009). Is the incidence of invasive vulvar cancer increasing in the United States?. Cancer Causes Control.

[3] Koual M., Ngo C., Bonsang-Kitzis H., Deloménie M., Balaya V., Nguyen-Xuan H.-T., Nos C., Tournat H., Le Frère Belda M.-A., Hivelin M. (2020). Prise en Charge Chirurgicale du Cancer de la Vulve.

[4] Burger M., Hollema H., Emanuels A.G., Krans M., Pras E., Bouma J. (1995). The Importance of the Groin Node Status for the Survival of T1 and T2 Vulval Carcinoma Patients. Gynecol. Oncol..

[5] Bosquet J.G., Magrina J.F., Gaffey T.A., Hernandez J.L., Webb M.J., Cliby W.A., Podratz K.C. (2005). Long-term survival and disease recurrence in patients with primary squamous cell carcinoma of the vulva. Gynecol. Oncol..

[6] Sedlis A., Homesley H., Bundy B.N., Marshall R., Yordan E., Hacker N., Lee J.H., Whitney C. (1987). Positive groin lymph nodes in superficial squamous cell vulvar cancer. Am. J. Obstet. Gynecol..

[7] Mahner S., Jueckstock J., Hilpert F., Neuser P., Harter P., de Gregorio N., Hasenburg A., Sehouli J., Habermann A., Hillemanns P. (2015). Adjuvant therapy in lymph node–positive vulvar cancer: The AGO-CaRE-1 Study. J. Natl. Cancer Inst..

[8] Brincat M.R., Muscat Baron Y. (2017). Sentinel lymph node biopsy in the management of vulvar carcinoma: An evidence-based in-sight. Int. J. Gynecol. Cancer.

[9] Levenback C.F. (2008). How safe is sentinel lymph node biopsy in patients with vulvar cancer?. J. Clin. Oncol..

[10] Schnürch H.G., Ackermann S., Alt C.D., Barinoff J., Böing C., Dannecker C., Gieseking F., Günthert A., Hantschmann P., Horn L.C. (2016). Diagnosis, Therapy and Follow-up Care of Vulvar Cancer and its Precursors. Guideline of the DGGG and DKG (S2k-Level, AWMF Registry Number 015/059, November 2015). Geburtshilfe Frauenheilkd..

[11] Hampl M., Hantschmann P., Michels W., Hillemanns P. (2008). Validation of the accuracy of the sentinel lymph node procedure in patients with vulvar cancer: Results of a multicenter study in Germany. Gynecol. Oncol..

[12] te Grootenhuis N.C., Van Der Zee A.G.J., Van Doorn H.C., van der Velden J., Vergote I., Zanagnolo V., Baldwin P.J., Gaarenstroom K.N., Van Dorst E.B., Trum J.W. (2016). Sentinel nodes in vulvar cancer: Long-term follow-up of the GROningen INternational Study on Sentinel nodes in Vulvar cancer (GROINSS-V) I. Gynecol. Oncol..

[13] Van der Zee A.G., Oonk M.H., De Hullu J.A., Ansink A.C., Vergote I., Verheijen R.H., Maggioni A., Gaarenstroom K.N., Baldwin P.J., Van Dorst E.B. (2008). Sentinel node dissection is safe in the treatment of early-stage vulvar cancer. J. Clin. Oncol..

[14] Azaïs H., Pauphilet V., Belghiti J., Nikpayam M., Gonthier C., Maingon P., Conforti R., Uzan C., Canlorbe G. (2019). Update regarding the management of vulvar cancer: The guidelines of the Assistance Publique–Hôpitaux de Paris. Bull. Cancer.

[15] Hassanzade M., Attaran M., Treglia G., Yousefi Z., Sadeghi R. (2013). Lymphatic mapping and sentinel node biopsy in squamous cell carcinoma of the vulva: Systematic review and meta-analysis of the literature. Gynecol. Oncol..

[16] Knopp S., Nesland J.M., Tropé C. (2008). SLNB and the importance of micrometastases in vulvar squamous cell carcinoma. Surg. Oncol..

[17] CNGOF (2005). Collège National des Gynécologues et Obstétriciens Français. J. Gynecol. Obstet. Biol. Reprod..

[18] Meads C., Sutton A.J., Rosenthal A.N., Małysiak S., Kowalska M., Zapalska A., Rogozińska E., Baldwin P., Ganesan R., Borowiack E. (2014). Sentinel lymph node biopsy in vulval cancer: Systematic review and meta-analysis. Br. J. Cancer.

[19] Wilke L.G., McCall L.M., Posther K.E., Whitworth P.W., Reintgen D.S., Leitch A.M., Gabram S.G.A., Lucci A., Cox C.E., Hunt K.K. (2006). Surgical complications associated with sentinel lymph node biopsy: Results from a prospective international cooperative group trial. Ann. Surg. Oncol..

[20] Vahrmeijer A.L., Hutteman M., van der Vorst J.R., van de Velde C.J.H., Frangioni J.V. (2013). Image-guided cancer surgery using near-infrared fluorescence. Nat. Rev. Clin. Oncol..

[21] Zapardiel I., Alvarez J., Barahona M., Barri P., Boldo A., Bresco P., Gasca I., Jaunarena I., Kucukmetin A., Mancebo G. (2021). Utility of intraoperative fluorescence imaging in gynecologic surgery: Systematic review and consensus statement. Ann. Surg. Oncol..

[22] Schaafsma B.E., Mieog J.S.D., Hutteman M., van der Vorst J.R., Kuppen P., Löwik C.W., Frangioni J.V., Van De Velde C.J., Vahrmeijer A.L. (2011). The clinical use of indocyanine green as a near-infrared fluorescent contrast agent for image-guided oncologic surgery. J. Surg. Oncol..

[23] Frangioni J.V. (2003). In vivo near-infrared fluorescence imaging. Curr. Opin. Chem. Biol..

[24] Vermersch C., Raia Barjat T., Perrot M., Lima S., Chauleur C. (2016). Place of indocyanine green coupled with fluorescence imaging in research of breast cancer sentinel node. Bull. Cancer.

[25] Papadia A., Mueller M.D. (2018). ICG-Enhanced Fluorescence-Guided SLN Mapping in Gynecological Malignancies.

[26] Soergel P., Hertel H., Nacke A.K., Klapdor R., Derlin T., Hillemanns P. (2017). Sentinel Lymphadenectomy in Vulvar Cancer Using Near-Infrared Fluorescence from Indocyanine Green Compared with Technetium 99m Nanocolloid. Int. J. Gynecol. Cancer.

[27] Verbeek F.P., Tummers Q.R., Rietbergen D.D., Peters A.A., Schaafsma B.E., Van De Velde C.J., Frangioni J.V., van Leeuwen F., Gaarenstroom K.N., Vahrmeijer A.L. (2015). Sentinel Lymph Node Biopsy in Vulvar Cancer Using Combined Radioactive and Fluorescence Guidance. Int. J. Gynecol. Cancer.

[28] Hutteman M., Van Der Vorst J.R., Gaarenstroom K.N., Peters A.A., Mieog J.S.D., Schaafsma B.E., Löwik C.W., Frangioni J.V., Van De Velde C.J., Vahrmeijer A.L. (2012). Optimization of near-infrared fluorescent sentinel lymph node mapping for vulvar cancer. Am. J. Obstet. Gynecol..

[29] Crane L.M.A., Themelis G., Arts H.J.G., Buddingh K.T., Brouwers A.H., Ntziachristos V., Van Dam G.M., Van Der Zee A.G.J. (2011). Intraoperative near-infrared fluorescence imaging for sentinel lymph node detection in vulvar cancer: First clinical results. Gynecol. Oncol..

[30] Schaafsma B.E., Verbeek F.P., Peters A.A., Van Der Vorst J., De Kroon C.D., Van Poelgeest M.I.E., Trimbos J.B.M., Van De Velde C.J., Frangioni J.V., Vahrmeijer A.L. (2013). Near-infrared fluorescence sentinel lymph node biopsy in vulvar cancer: A randomised comparison of lymphatic tracers. BJOG Int. J. Obstet. Gynaecol..

[31] Mathéron H., Berg N.V.D., Brouwer O., KleinJan G., van Driel W.J., Trum J., Vegt E., Kenter G., van Leeuwen F., Olmos R.V. (2013). Multimodal surgical guidance towards the sentinel node in vulvar cancer. Gynecol. Oncol..

[32] Prader S., du Bois A., Harter P., Breit E., Schneider S., Baert T., Heitz F., Traut A., Ehmann S., Pauly N. (2020). Sentinel lymph node mapping with fluorescent and radio-active tracers in vulvar cancer patients. Arch. Gynecol. Obstet..

[33] Broach V., Abu-Rustum N.R., Sonoda Y., Brown C.L., Jewell E., Gardner G., Chi D.S., Zivanovic O., Leitao M.M. (2020). Evolution and outcomes of sentinel lymph node mapping in vulvar cancer. Int. J. Gynecol. Cancer.

[34] Deken M.M., van Doorn H.C., Verver D., Boogerd L.S., de Valk K.S., Rietbergen D.D., van Poelgeest M.I., de Kroon C.D., Beltman J.J., van Leeuwen F.W. (2020). Near-infrared fluorescence imaging compared to standard sentinel lymph node detection with blue dye in patients with vulvar cancer—A randomized con-trolled trial. Gynecol. Oncol..

[35] Crosbie E.J., Winter-Roach B., Sengupta P., Sikand K.A., Carrington B., Murby B., Slade R.J. (2010). The accuracy of the sentinel node proce-dure after excision biopsy in squamous cell carcinoma of the vulva. Surg. Oncol..

[36] Milliken S., May J., Sanderson P.A., Congiu M.A., D’Oria O., Caruso G., Di Donato V., Giannini A. (2021). Reducing the radicality of surgery for vulvar cancer: Are smaller margins safer?. Minerva Gynecol..

[37] Giannini A., D’Oria O., Chiofalo B., Bruno V., Baiocco E., Mancini E., Mancari R., Vincenzoni C., Cutillo G., Vizza E. (2021). The giant steps in surgical downsizing toward a personalized treatment of vulvar cancer. J. Obstet. Gynaecol. Res..

